# Genome-Wide Association Mapping and Genomic Prediction Analyses Reveal the Genetic Architecture of Grain Yield and Flowering Time Under Drought and Heat Stress Conditions in Maize

**DOI:** 10.3389/fpls.2018.01919

**Published:** 2019-01-30

**Authors:** Yibing Yuan, Jill E. Cairns, Raman Babu, Manje Gowda, Dan Makumbi, Cosmos Magorokosho, Ao Zhang, Yubo Liu, Nan Wang, Zhuanfang Hao, Felix San Vicente, Michael S. Olsen, Boddupalli M. Prasanna, Yanli Lu, Xuecai Zhang

**Affiliations:** ^1^Maize Research Institute, Sichuan Agricultural University, Wenjiang, China; ^2^Key Laboratory of Biology and Genetic Improvement of Maize in Southwest Region, Ministry of Agriculture, Chengdu, China; ^3^International Maize and Wheat Improvement Center, Texcoco, Mexico; ^4^International Maize and Wheat Improvement Center, Harare, Zimbabwe; ^5^International Maize and Wheat Improvement Center, Nairobi, Kenya; ^6^College of Bioscience and Biotechnology, Shenyang Agricultural University, Shenyang, China; ^7^Institute of Crop Sciences, Chinese Academy of Agricultural Sciences, Beijing, China

**Keywords:** maize, association mapping, genomic prediction, drought stress, heat stress, combined drought and heat stress

## Abstract

Drought stress (DS) is a major constraint to maize yield production. Heat stress (HS) alone and in combination with DS are likely to become the increasing constraints. Association mapping and genomic prediction (GP) analyses were conducted in a collection of 300 tropical and subtropical maize inbred lines to reveal the genetic architecture of grain yield and flowering time under well-watered (WW), DS, HS, and combined DS and HS conditions. Out of the 381,165 genotyping-by-sequencing SNPs, 1549 SNPs were significantly associated with all the 12 trait-environment combinations, the average PVE (phenotypic variation explained) by these SNPs was 4.33%, and 541 of them had a PVE value greater than 5%. These significant associations were clustered into 446 genomic regions with a window size of 20 Mb per region, and 673 candidate genes containing the significantly associated SNPs were identified. In addition, 33 hotspots were identified for 12 trait-environment combinations and most were located on chromosomes 1 and 8. Compared with single SNP-based association mapping, the haplotype-based associated mapping detected fewer number of significant associations and candidate genes with higher PVE values. All the 688 candidate genes were enriched into 15 gene ontology terms, and 46 candidate genes showed significant differential expression under the WW and DS conditions. Association mapping results identified few overlapped significant markers and candidate genes for the same traits evaluated under different managements, indicating the genetic divergence between the individual stress tolerance and the combined drought and HS tolerance. The GP accuracies obtained from the marker-trait associated SNPs were relatively higher than those obtained from the genome-wide SNPs for most of the target traits. The genetic architecture information of the grain yield and flowering time revealed in this study, and the genomic regions identified for the different trait-environment combinations are useful in accelerating the efforts on rapid development of the stress-tolerant maize germplasm through marker-assisted selection and/or genomic selection.

## Introduction

Maize is the major source of food security and economic development in the major developing countries in sub-Saharan Africa, Latin America and Asia (Cairns and Prasanna, 2018). Drought stress (DS) has long been recognized as a major constraint to maize yield production in these regions, which affects approximately 20% of the tropical and subtropical maize in any given year in the developing countries (Heisey and Edmeades, 1999; Cairns et al., 2013a; Cerrudo et al., 2018). Climate projections suggest decreasing precipitation, increasing temperatures, and a higher intensity and frequency of extreme events. Heat stress (HS) alone and in combination with DS are likely to become the increasing constraints to maize production in the region of maize-dependent countries (Cairns et al., 2013a). This highlight the need to develop and deploy climate-resilient maize varieties in the tropical world.

While genetic gain for grain yield (under experimental conditions) under DS in sub-Saharan Africa is similar to other regions of the world, absolute yields in farmers’ fields remain low (Masuka et al., 2017a,b). Breeding for HS in maize in sub-Saharan Africa was only initiated in 2011 and, to date, genetic gain in grain yield has not been quantified under HS. Increasing genetic gain for yield under climate related stresses will be an important component of offsetting future losses under climate change. Lead times for maize breeding in sub-Saharan Africa are currently too slow to adapt to climate change (Challinor et al., 2016; Atlin et al., 2017). Molecular breeding offers the ability to expand the size of a breeding program, thereby increasing selection intensity, without increasing phenotyping requirements (Olsen et al., unpublished). Genotypic information can be used to select germplasm prior to the phenotyping stages and the capability to increase this phenotypically untested layer will allow the total number of genotypes within a breeding program to be expanded (Cooper et al., 2014). Understanding the genetic architecture of DS or HS tolerance alone or in a combination, by identifying and validating genomic regions conferring tolerance to stresses and developing production molecular markers can significantly accelerate the development of stress-resilient maize varieties through marker-assisted selection or genomic selection (GS).

The genetic studies for understanding the genetic architecture of DS tolerance in maize have been conducted on grain yield (GY) and secondary traits with strong genetic correlation with GY over a wide range of genetic mapping populations with different population sizes, marker types, and marker densities, where the phenotypic data were collected from the inbred lines and their testcross hybrids (Hao et al., 2010; Semagn et al., 2013;Cerrudo et al., 2018). These studies showed that maize is highly susceptible to DS during flowing and early grain filling stages, secondary traits including anthesis date (AD) and anthesis silking interval (ASI) are always with strong genetic correlation with GY and these traits are highly heritable and cost-effective to measure, which are potential to be included in the breeding program to facilitate indirect selection for GY.

The quantitative trait loci (QTL) conferring tolerance to DS in maize were investigated in several linkage mapping studies to reveal the genetic architecture of the GY and the secondary traits (Lu et al., 2006; Hao et al., 2010; Semagn et al., 2013; Cerrudo et al., 2018). Lu et al. (2006) mapped 21 additive QTL and 61 epistatic QTL pairs in a recombinant inbred line populations using 261 simple sequence repeat markers, these QTL significantly associated with the GY and the secondary traits under well-watered (WW) and DS conditions. In a F_2_ population consisted of 234 individuals, two consensus QTL controlling GY and the secondary traits were identified by Hao et al. (2008) in bin 1.03 and bin 9.03 – 9.05, where 130 simple sequence repeat markers and 190 amplified fragment length polymorphism markers were used for genotyping. Recently, Cerrudo et al. (2018) detected new QTL regions associated with GY and the secondary traits in bins 1.02 and 1.03 in a bi-parental doubled haploid population. In addition to individual population-based linkage mapping, few meta-analyses were also performed in multiple bi-parental populations. A meta-analysis in twelve bi-parental populations revealed 39 and 36 consensus QTL significantly associated with GY and secondary traits under DS and WW condition, respectively (Hao et al., 2010). In another meta analyses study with 18 populations, Semagn et al. (2013) found 68 consensus QTL significantly associated with GY and the secondary traits. Compared with individual population-based linkage mapping analysis, the meta-analysis reduced the number of QTL detected by 68% and increased the mapping resolution by 12-fold (Semagn et al., 2013).

Genome-wide association study (GWAS) in a panel consisted of 350 tropical and subtropical maize inbred lines using 57647 SNPs revealed 33 candidate genes associated with GY and the secondary traits for WW and DS conditions(Xue et al., 2013). Thirunavukkarasu et al. (2014) detected 52 candidate genes in another GWAS panel consisted of 240 maize inbred lines, which were associated with seven agronomic traits including GY and the secondary traits evaluated under WW and DS conditions. Moreover, GWAS were also conducted in panels of multiple bi-parental populations. Li et al. (2016) conducted association analysis in a panel consisted of 5000 nested association mapping recombinant inbred lines. In total, 169 QTL and 220 QTL were detected, which significantly associated with the seven target traits under DS and WW condition, respectively. Similarly, Wallace et al. (2016) also conducted association mapping in fifteen tropical maize bi-parental populations to reveal the genetic architecture of plant height and flowering time evaluated under WW and DS conditions. In addition, joint linkage-association mapping approach was used for improving mapping power and resolution for revealing the genetic architecture of DS tolerance in maize. Lu et al. (2010) performed joint linkage-association mapping in three recombinant inbred line populations and a panel consisted of 305 inbred lines, where the QTL detection power and mapping resolution were improved, and the results also suggested that the haplotype-based method improved the QTL detection power of association mapping.

Candidate genes identified from the genetic studies are possible to be validated by expression profiling analysis with RNA-seq data. Xu et al. (2014) validated 262 out of the 271 DS response candidate genes using RNA-seq data, these candidate genes expressed differentially between the drought tolerant line and the drought sensitive line, which were treated under different water stress conditions. Li et al. (2016) also validated 52 of the 354 DS tolerance relevant candidate genes in another study, these candidate genes showed significant differential expression in the inbred lines B73 under the WW and DS conditions.

Genomic selection, also named as genomic prediction (GP), incorporates all available marker information into a prediction model to predict the genomic estimated breeding values of the unknown-phenotype breeding materials for selection, where all the alleles with both major effects and minor effects are captured simultaneously (Meuwissen et al., 2001). Therefore, GS is an effective approach to improve the complex traits, several studies showed the effectiveness of implementing GS for DS tolerance improvement in maize (Beyene et al., 2015; Zhang et al., 2015; Vivek et al., 2016; Wallace et al., 2016; Zhang A. et al., 2017; Zhang X. et al., 2017). Low to medium prediction accuracy were reported in maize on GY and the secondary traits under DS, where the results suggested that the prediction accuracy values were mainly affected by breeding population types, training population size, trait complexities, marker densities, and genotyping platforms (Zhang et al., 2015). Incorporating known marker-trait associations into prediction model is also beneficial to increase the prediction accuracy. In fifteen bi-parental maize populations, Wallace et al. (2016) showed that the SNPs identified from the association mapping analysis had prediction accuracies equivalent to the genome-wide SNPs for plant height and flowering time under DS and WW conditions.

In contrast to intensive genetic studies on DS tolerance in maize, relatively less effort has been made for HS tolerance, and the tolerance to combined drought and heat stress (Cairns et al., 2013a). Most of the HS research is focused on high yield production in temperate maize germplasm. Efforts on understanding the tolerance to HS and tolerance to combined drought and heat stress (DHS) in tropical and subtropical maize has been initiated recently (Cairns et al., 2013a; Alam et al., 2017). In this study, the DTMA (Drought Tolerant Maize for Africa) association-mapping panel including 300 tropical maize inbred lines was genotyped with genotyping-by-sequencing (GBS), and the testcross performance of these maize inbred lines were evaluated in multi-environment trials under the conditions of WW, managed DS, HS, and DHS. The main objectives of this study are: (1): to perform the GWAS by single-SNP-based and haplotype-based methods to reveal the genetic architecture of GY, AD and ASI under different managements conditions; (2) to identify the marker-trait associations SNPs and candidate genes for all the trait-environment combinations, and validate the candidate genes with expression profiling analysis using the RNA-seq data; (3) to predict the performance of lines for GY, AD and ASI under different management conditions with genome-wide high density SNPs and marker-trait associated SNPs, and compare their prediction accuracies.

## Materials and Methods

### Plant Materials and Experimental Management

The DTMA panel consisted of 300 tropical and subtropical maize inbred lines was used in this study for association mapping and GP analyses (Wen et al., 2011; Cairns et al., 2013a). The testcrosses were formed by crossing each of the inbred line in the panel with the tester CML539, a broadly adapted tropical maize inbred line. In total, 15 multiple environment trials were performed at the field experimental stations in Mexico, Kenya, Thailand, Zimbabwe and India in 2008–2011 as previously described (Cairns et al., 2013a) under conditions of WW, managed DS, HS, and DHS. Briefly, experiments were planted during the dry season in all locations except for India to ensure DS to be imposed at the anthesis stage. The α-lattice experimental design was applied with three replicates in 2009 and two replicates in 2010 and 2011. The managed DHS condition was with daily maximum temperatures exceeded 35°C for more than 30d and vapor pressure deficit was very low compared to individual HS condition during the reproductive stage.

The target traits in the current study are GY, AD, and ASI. In total, 12 trait-environment combinations between the three target traits and the four evaluation conditions were investigated. For each of the target trait evaluated under the different conditions, the estimated best linear unbiased prediction values (Cairns et al., 2013a) were applied to the following analyses. More details on phenotypic analyses of these traits were previously described (Cairns et al., 2013a). The phenotypic data of the estimated best linear unbiased prediction values is available from the following repository: http://hdl.handle.net/11529/10548156. The Pearson’s correlation coefficients were calculated by using R function *cor.test ()* and the pair-wise correlations were visualized with function *pairs ()*.^1^

### GBS SNPs

A GBS protocol developed by Elshire et al. (2011) was applied in this study, which is commonly used by the maize research community. For each of the inbred line in the DTMA panel, the total genomic DNA was extracted from the bulked young leaves with a CTAB method (CIMMYT Applied Molecular Genetics Laboratory, 2003). The genotyping work was performed at the Biotechnology Resource Center of Cornell University (Ithaca, NY, United States) by multiplexing 96 samples on each sequencing lane. The SNP calling was performed following the TASSEL GBS workflow, and the GBS 2.7 TOPM (tags on physical map) file downloaded from Panzea ^2^ was used to anchor reads to the B73 reference genome (Elshire et al., 2011; Glaubitz et al., 2014; Wu et al., 2016). In total, 955,690 SNPs were generated and the imputation of the missing markers were performed with FILLIN method by accepting the default parameters (Swarts et al., 2014; Cao et al., 2017). The SNP data is available from the following repository: http://hdl.handle.net/11529/10548156.

### SNP-Based Association Mapping and Candidate Gene Annotation

The imputed SNP dataset was filtered with minor allele frequency greater than 1% and a missing rate less than 25%. The SNPs with known physical position and good quality were subjected to further analysis. The filtered SNP dataset was used to perform the GWAS, where the associations between the SNPs and the interested traits were detected by employing the R package Genome Association and Prediction Integrated Tool (GAPIT) (Lipka et al., 2012). In order to control the false associations and to solve the computational problem, the SUPER (Settlement of MLM Under Progressively Exclusive Relationship) method integrated in GAPIT, was employed to perform the association mapping analysis by incorporating the population structure analysis and the relative kinship matrix (Wang et al., 2014). The principal component analysis was used to stratify the population structure, and the relative kinship matrix was used to assess the relatedness among individuals in the association mapping panel. The first eight principal components estimated in GAPIT was used to stratify the population structure, and a subset of SNPs extracted by SUPER method was used to calculate kinship matrix by assessing the relatedness among individuals in the DTMA panel. The parameter of “*sangwich.top*” and “*sangwich.bottom*” was set as *MLM* and *SUPER*, respectively. Compared with the FaST-LMM (Factored Spectrally Transformed Linear Mixed Model), the SUPER method not only retains the computational advantage, but also increases statistical power (Lippert et al., 2011; Wang et al., 2014).

A moderate *p*-value with a threshold of 1 × 10^-4^ was used to declare the significant associations. The quantile–quantile plot and Manhattan plot generated with customized R scripts were used to visualize the observed P (-log_10_ *^p^*^-values^) of SNP-trait associations. The candidate genes were identified as the genes that the significant SNPs located in or adjacent to (<3kb) against B73 AGPV2 (MaizeGDB)^3^. The significant SNPs and candidate genes were visualized using R package “VennDiagram” for comparison. The significantly associated SNPs were then grouped in genomic regions with sliding window method (window size = 20 Mb) from the first significant SNPs on each chromosome for each trait. The hotspot regions were identified as the top 5% quantile of density of significant SNPs for each trait.

### Haplotype-Based Association Mapping

The gene-based-haplotype method was applied to perform association analysis as well, where each gene was regarded as a window and the SNPs within each gene were used to construct the haplotypes (Ding et al., 2015). Only the genes containing at least two SNPs were included into the analysis, five randomly selected SNPs of each gene were used to build the haplotypes, when the target genes contain more than five SNPs (up to 81 SNPs within GRMZM2G002559). In total, 301,897 haplotypes were built on the 19,674 annotated genes, with an average of 15 haplotypes/gene. The haplotypes were filtered with frequency greater than 5% and missing rate less than 25%. A haplotype-based association mapping was carried out in TASSEL 3.0^4^, where the principal component and the kinship matrix calculated with the filtered SNP dataset above were incorporated into the MLM model. A moderate *p*-value with a threshold of 1 × 10^-4^ was used to declare the significant.

### Functional Annotation and Expression Profiling Analysis of the Candidate Genes

For functional annotation analysis, the DNA sequence information of the candidate genes identified from the association mapping analyses was submitted to NCBI^5^ and Phytozome^6^ to search the best match. The summarized flowering time related genes and the reported loci underlying related traits were collected in this study to evaluate the association results (Wallace et al., 2016). Simultaneously, information of the candidate genes were submitted to the web-based tool AgriGO^7^ to perform gene ontology-based functional enrichment analysis (Xu et al., 2014). Briefly, singular enrichment analysis tool was selected to carry out GO (gene ontology) enrichment analysis, and the Fisher’s exact test coupled with the Bonferroni for multi-test adjustment method (FDR < 0.05) was used to select enrichment GO terms.

Expression profiling analysis was conducted by using the transcriptomic sequencing data of four tropical maize inbred lines as previously described (Xu et al., 2017). The four inbred lines including one drought tolerant line (AC7643), one drought sensitive line (AC7729/TZSRW), one drought tolerant RIL (RIL208) and one drought sensitive RIL (RIL64) derived from the above two parental lines, were cultivated in nutrient solution under simulated DS and WW conditions. After treated with 10% (w/v) polyethylene glycol PEG 8000 (Sigma-Aldrich) for 24 h at the three-leaf stage, the simulated DS condition, the roots from three seedling plants for each of the four inbred lines under different conditions were collected for RNA extraction separately using TRIzol^®^ reagent (Invitrogen, United States) (Xu et al., 2017). A cutoff of the absolute value of fold-change > 2(|log_2_^fold-change^| > 1) and *q*-value ≤ 0.01 (1% FDR) (Trapnell et al., 2013) of the ratio of expression levels under DS *vs.* WW for each candidate genes, at least in one line, were used to determine whether the candidate genes were differentially expressed genes (DEGs) or DS responsive genes. The raw RNA sequencing data is available from the following repository in the NCBI Sequence Read Archive under accession number PRJNA294848 (SRP063383): https://www.ncbi.nlm.nih.gov/bioproject/?term=PRJNA294848.

### Genomic Prediction

Genomic prediction was performed by using rrBLUP model, implemented in R with rrBLUP package for all the traits evaluated under different conditions (Endelman, 2011). Prediction accuracies of the complex traits were estimated from the genome-wide markers and the significant SNPs. Two kinds of marker density were applied for GP analyses. A genome-wide marker dataset including 10,108 high quality SNPs with minor allele frequency greater than 5 and 0% of missing, were selected to perform the GP for all the traits. In parallel, the significantly associated SNPs detected in the SNP-based association mapping analyses, were used to perform the GP for each traits. A five-fold cross-validation scheme with 100 replications was used to generate the training and validation subsets and assess the prediction accuracy for each trait. The average value of the correlations between the phenotypic and the genomic estimated breeding values was defined as the *r_MG_* to assess the prediction accuracy.

## Results

### Phenotypic Variation and Correlation Among the Target Traits

**FIGURE 1:**
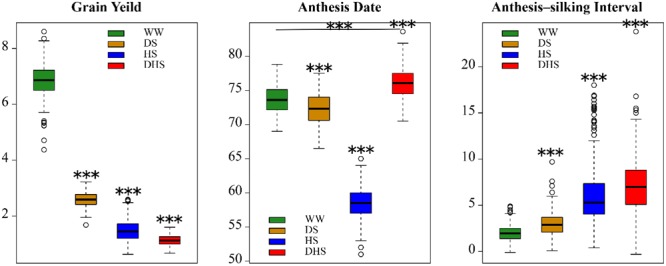
The boxplots of the phenotypic distribution of all the 12 target trait-environment combinations between the three target traits, i.e., grain yield (ton/hectare); anthesis date (day); anthesis-silking interval (day), and the four evaluation conditions, i.e., well-watered (WW), drought stress (DS), heat stress (HS), and combined drought and heat stress (DHS).

For each of the target traits, the range of phenotypic distribution was large under each management, indicating that the broader diversity in the panel (Figure 1). For each trait, the mean performance and the range was varied with different management conditions (Figure 1). The mean performance for all the target traits evaluated under WW conditions were significantly different with those evaluated under the stress conditions. The mean value and the phenotypic distribution of GY evaluated under WW condition were greater than those evaluated under the stress conditions. However, the phenotypic variations for AD and ASI evaluated under WW condition were narrow than those evaluated under the stress conditions. The mean value of AD evaluated under WW condition was greater than that evaluated under the stress conditions, with an exception of DHS management. Nevertheless, the mean value of ASI evaluated under WW condition was lower than that evaluated under the stress conditions. Broad sense heritability for all the target traits evaluated under different conditions were moderate to high (Cairns et al., 2013b). This suggests that the managed stress tolerance evaluation in this study is effective and reliable.

**FIGURE 2:**
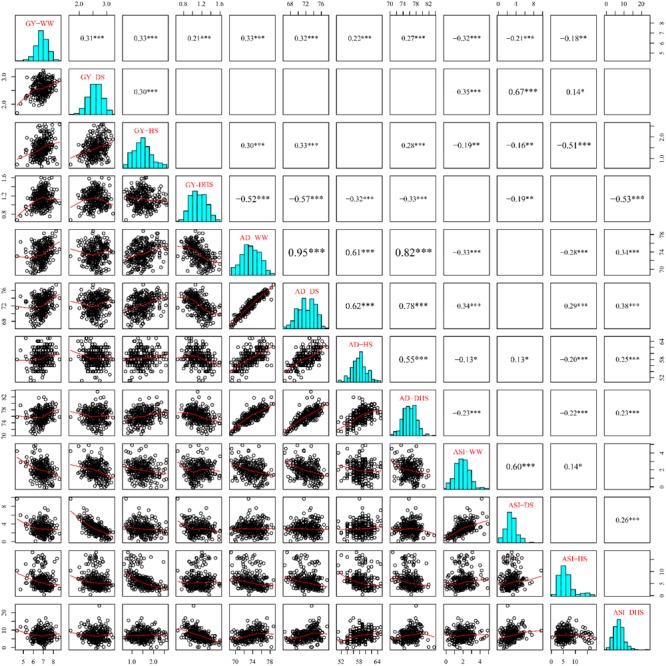
The pairwise correlations among the 12 trait-environment combinations based on the BLUP values. The traits of GY, AD and ASI are abbreviated from grain yield, anthesis date and anthesis–silking interval, respectively. The environments of WW, DS, HS, and DHS are abbreviated from well-watered, drought stress, heat stress and combined drought and heat stress management conditions. The numbers with one or more star(s) represents the Pearson correlation coefficients at different significances (^∗^: 0.05; ^∗∗^: 0.01; ^∗∗∗^: 0.001) and the word size of them indicate the correlation level. The blank boxes indicate that there was no significant correlation for the corresponding traits.

The phenotypic correlations differed among the same trait evaluated under the different management conditions (Figure 2). The phenotypic correlations for GY under different management conditions were moderate, positive and significant except for DHS with DS and HS (Figure 2). For AD, the correlations were positive and highly significant between all management conditions. For ASI, phenotypic correlations were significant only with WW, DS, and HS, whereas ASI under DHS was significantly correlated with DS. Other combinations of stress were not significant. The phenotypic correlations of the same trait evaluated under the different conditions depend on the complexity level of the target traits. For the less complex trait like AD, all the correlations among the different conditions were ranged from 0.55 to 0.95. For the complex traits like GY and ASI evaluated under the different conditions, most of the correlations were ranged from 0.14 to 0.60. However, GY evaluated under DHS condition was not correlated significantly with either of the traits evaluated under DS or HS condition, the indirect relationships were observed for GY evaluated under the DHS with that evaluated under the single stress condition (DS or HS). The ASI evaluated under WW condition was positively correlated with that evaluated under the single stress condition (DS or HS), and the ASI evaluated under the HS condition was not correlated with that evaluated under the DS and DH conditions. It meant that ASI is also a very complex trait.

The phenotypic correlations also differed among the different target traits evaluated under the same condition. In general, the GY was negatively and significantly correlated with ASI significantly under each evaluation condition with a range of -0.32 to -0.67, this is consistent with the previous observation (Ribaut et al., 2009). However, the consistent correlations were not observed between GY and AD, and between AD and ASI evaluated under the same condition.

### The SNP-Based Genome-Wide Associations

**FIGURE 3:**
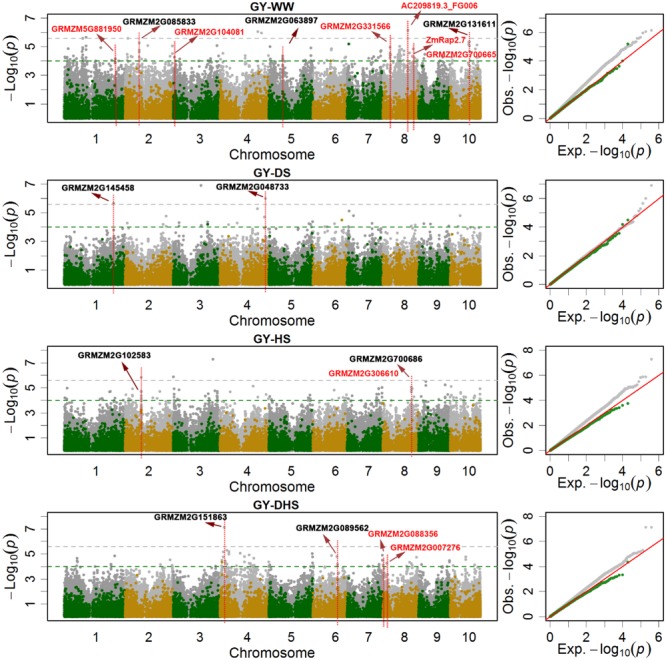
The Manhattan plots and Q-Q plots of the SNP-based (gray dots) and the haplotype-based (green and yellow dots) association mapping for grain yield (GY) under different conditions. WW, DS, HS, and DHS are abbreviated from well-watered, drought stress, heat stress and combined drought and heat stress management conditions. The red colored candidate genes are the previously reported, and the blank colored are novel candidate genes.

In total, 381,165 filtered SNPs were used to perform the GWAS for the 12 trait-environment combinations. Number of the significantly associated SNPs of each trait-environment combination ranged from 8 for ASI-HS to 335 for AD-DS, and 1661 associations in total were identified with the p-value threshold of 1 × 10^-4^ for all the 12 trait-environment combinations. The average PVE for 1661 associations was 4.33% and 589 of them had a PVE value greater than 5% (Table 1 and Supplementary Table S1). At the *p*-value threshold of 2.6 × 10^-6^ (1/n, n indicates the number of SNPs), 83 associations in total were identified for all the 12 trait-environment combinations, except for ASI-HS and ASI-DH. Number of significantly associated SNPs identified above the *p*-value threshold of 2.6 × 10^-6^ ranged from 2 for GY-DH and AD-DH to 32 for AD-DS (Table 1, Supplementary Table S1, Figure 3, and Supplementary Figures S1, S2). The average PVE value of these 83 associations was 4.88% and 5 of them had a PVE value greater than 10%.

**Table 1:** The genome-wide association mapping results of the 12 trait-environment combinations in the DTMA panel using 381,165 filtered SNPs.

Trait-					No. of candidate	No. of significant	No. of
Environment^∗^	No. of significant SNPs				genes	genomic region of 20 Mb	SNPs per region
				
	*p* < 10^-4^	*R*^2^> 5%	*p* < 2.6 × 10^-6^	in genic region			
GY-WW	191	88	5	126	82	47	4.06
GY-DS	36	9	3	20	17	22	1.64
GY-HS	150	37	4	89	55	40	3.75
GY-DHS	76	17	2	50	41	30	2.53
AD-WW	274	112	5	167	128	64	4.28
AD-DS	335	100	32	225	147	61	5.49
AD-HS	69	24	2	51	36	28	2.46
AD-DHS	302	107	22	189	132	56	5.39
ASI-WW	53	21	5	24	18	25	2.12
ASI-DS	101	46	3	65	45	40	2.53
ASI-HS	8	0	0	6	6	7	1.14
ASI-DHS	66	25	0	39	27	26	2.54
**Total**	**1661**	**586**	**83**	**1038**	**734**	**446**	**3.72**

*^∗^The traits of GY, AD, and ASI are abbreviated from grain yield, anthesis date and anthesis–silking interval, respectively. The environments of WW, DS, HS, and DHS are abbreviated from well-watered, drought stress, heat stress and combined drought and heat stress management conditions.*

**FIGURE 4:**
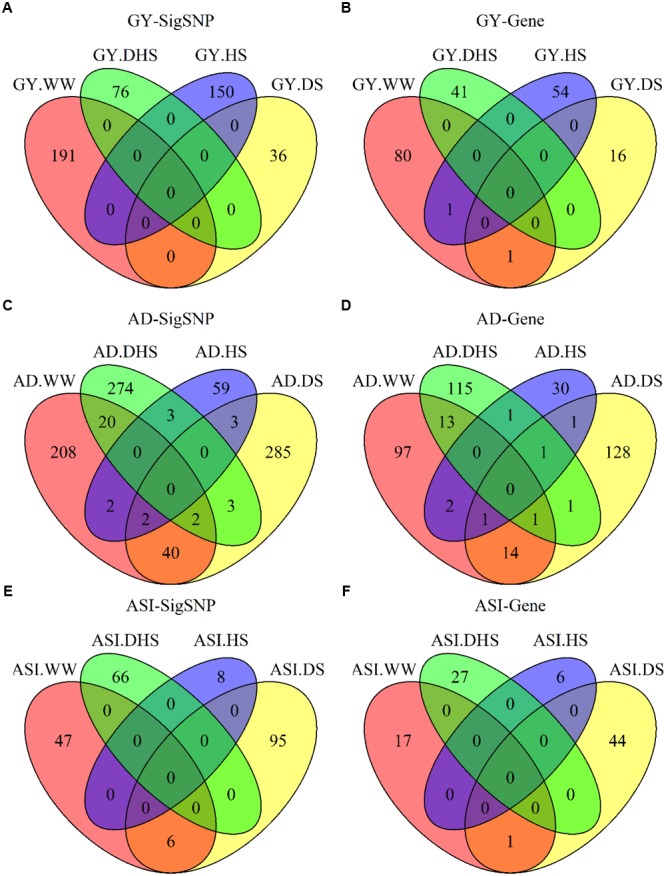
The number of overlapping SNPs **(A,C,E)** and candidate genes **(B,D,F)** for each trait evaluated under different conditions. GY, AD, and ASI indicate grain yield, anthesis date, and anthesis–silking interval, respectively. WW, DS, HS, and DHS are abbreviated from well-watered, drought stress, heat stress and combined drought and heat stress management conditions.

The number of overlap SNPs for the same trait evaluated under the different conditions showed in Figure 4. The analysis of the number of overlap SNPs was highly consistent with the phenotypic analysis. Number of the overlap SNPs between different conditions related with the complexity level of the target traits. For the less complex trait like AD evaluated under the different conditions, number of the overlap SNPs ranged from 0 to 40, the maximum number of overlap SNPs were observed between WW and DS conditions. For ASI, only two overlap SNPs were observed between WW and DS conditions. For GY, no overlap SNPs were observed among all the evaluation conditions. This information indicated the genetic divergence between the individual stress tolerance and the combined drought and heat stress tolerance.

Genomic regions significantly associated with the target trait were identified by using a sliding window size of 20 Mb on each chromosome by merging the neighbor SNPs. In total, 446 genomic regions were identified for all the 12 trait-environment combinations. The numbers of genomic regions associated with the target traits were ranged from 7 for ASI-HS to 64 for AD-WW. Number of genomic regions identified for GY evaluated under WW, DS, HS and DHS conditions were 47, 22, 40, and 30 with the SNP density of 4.06, 1.64, 3.75, and 2.53 per genomic region, respectively. Compared to GY, more genomic regions and higher SNP densities were found for AD except for AD-HS, and less genomic regions and lower SNP densities were identified for ASI except for ASI-HS evaluated under the same condition (Table 1 and Supplementary Table S1). For the 12 trait-environment combinations, 33 hotspot regions were identified, and the number of hotspot region for each trait-environment combination ranged from one to four (Supplementary Table S2 and Supplementary Figure S3). For GY, the hotspot regions were distributed on chromosomes 1, 2, 4, 5, 8 and 10. The largest number of hotspot regions were observed on chromosome 8, which harbored eleven hotspot regions significantly associated with GY evaluated under different conditions. The second largest number of hotspots were observed on chromosome 1, which harbored six hotspot regions for GY.

### The Haplotype-Based Genome-Wide Associations

In total, 301,897 haplotypes formed from the 19,674 annotated genes were used to perform the haplotype-based association mapping. Compared with the SNP-based association mapping method, the haplotype-based associated mapping method detected fewer number of significantly associated SNPs with higher PVE values (Table 2 and Supplementary Table S3). At the significant threshold (*p* < 1 × 10^-4^), 311 haplotypes associated for all the target trait-environment combinations, except for the three traits under HS condition, i.e., GY-HS, AD-HS, ASI-HS were detected. Number of significantly associated haplotypes for each trait-environment combination ranged from 3 for ASI-WW to 26 for both AD-DS and GY-WW. The average PVE values was 10.56% with a range from 5.63 to 19.71%, which was higher than that in the SNP-based association mapping analysis.

**Table 2:** The haplotype-based genome-wide association mapping results of the 12 trait-environment combinations in the DTMA panel.

Trait-	No. of significant	No. of genic	No. of candidate		Overlap candidate gene between haplotype-based
Environment^*^	haplotypes	SNPs	genes	Average *R*^2^	and SNP-based association mapping analyses
GY-WW	46	13	2	11.73%	
GY-DS	22	6	2	9.16%	
GY-HS	0	0	0	0.00%	
GY-DHS	6	2	1	10.03%	
AD-WW	34	23	2	11.04%	GRMZM2G109651; GRMZM2G313009
AD-DS	89	41	5	10.91%	GRMZM2G109651; GRMZM2G043764
AD-HS	0	0	0	0.00%	
AD-DHS	13	16	1	7.98%	
ASI-WW	3	4	1	6.70%	
ASI-DS	73	41	5	9.15%	GRMZM2G329229
ASI-HS	0	0	0	0.00%	
ASI-DHS	25	10	1	19.71%	
**Total**	**311**	**156**	**20**		

^*^The traits of GY, AD, and ASI are abbreviated from grain yield, anthesis date and anthesis–silking interval, respectively. The environments of WW, DS, HS, and DHS are abbreviated from well-watered, drought stress, heat stress and combined drought and heat stress management conditions.

### The Candidate Genes Associated With the Target Traits Evaluated Under Different Conditions

In the SNP-based association mapping analysis, 120 significant associations had a pleiotropy effects for at least two trait-environment combinations, 1038 significant associations were in the genic region of 673 candidate genes, and the average number of SNPs per candidate gene was 1.36, i.e., (1038-120)/673. The max number of SNPs per candidate gene, i.e., 13, observed for GRMZM2G700686 associated with trait-environment combination of GY-HS (Supplementary Table S1 and Figure 3, 4). Number of candidate genes differed among the 12 trait-environment combinations, more candidate genes were detected for the less complex traits. The total number of candidate genes detected for the target trait across all the evaluation conditions was 193, 95, and 405 for GY, ASI, and AD, respectively.

Few candidate genes overlapped for the same traits evaluated under the different conditions, indicating the genetic divergence between the individual stress tolerance and tolerance for DHS. Two candidate genes were overlapped for GY evaluated under the different conditions, i.e., one common candidate gene between WW and DS conditions, and one common candidate gene between WW and HS conditions. Only one overlap candidate gene was observed for ASI evaluated under WW and DS conditions. Number of the overlap genes for AD evaluated under the different conditions ranged from zero to 14, with the maximum number of genes overlapped for AD evaluated between WW and DS conditions.

In the haplotype-based association mapping analysis, 19 candidate genes were identified for the 12 trait-environment combinations, and 156 SNPs were in the genic region of these candidate genes, 87 SNPs were used to build the haplotypes of the candidate genes, the haplotypes of each candidate gene was built with two to five SNPs in the genic region. The number of the candidate genes for all the trait-environment combinations ranged from zero to five, and the total number of candidate genes detected for the target trait across all evaluation conditions was 5, 7, and 8 for GY, ASI, and AD, respectively.

Four candidate genes, i.e., GRMZM2G329229, GRMZM2G313009, GRMZM2G043764, and GRMZM2G10 9651, overlapped in both the SNP-based and the haplotype-based association mapping analyses (Table 2 and Supplementary Table S3). Three of them were associated with AD evaluated under different conditions, the other one was overlapped between GY-HS and ASI-DS. The PVE value of these four genes were > 9.13%. The relatively high PVE for these candidate genes indicated their major effect on trait expression.

### The Biological Metabolic Processes Involved by Candidate Genes and the Differentially Expressed Candidate Genes Under Drought Stress

**FIGURE 5:**
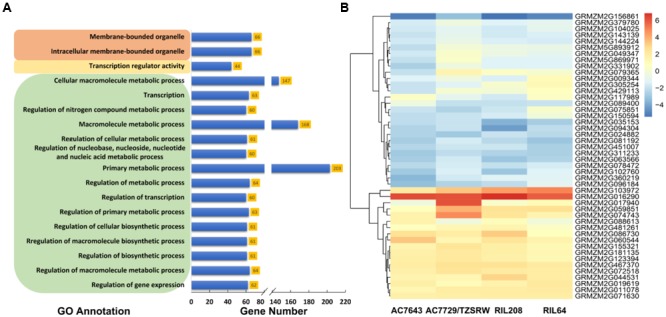
The results of gene ontology (GO)-based functional enrichment analysis **(A)** and the expression profiling analysis **(B)**. The GO terms in brown, yellow, and green colored boxes are cellular component, molecular function, and biological process categories, respectively. AC7643 and RIL208 are drought tolerance maize inbred lines, AC7729/TZSRW and RIL64 are drought sensitive maize inbred lines, and RIL 208 and RIL64 are derived from the cross of AC7643 and AC7729/TZSRW.

The results of gene ontology-based functional enrichment analysis shown that the 688 candidate genes were significantly (FDR < 0.05) enriched to 15 GO terms related with different biological processes (Figure 5A). Two terms, GO: 0043231 and GO: 0043227, were involved in cellular components. One term of GO: 0030528 was involved in the molecular function with transcription regulator activity (Figure 5A and Supplementary Figure S4). The biological process was mainly involved in metabolic process, including regulation of metabolic process (GO: 0019222), macromolecule metabolic process (GO: 0043170) and primary metabolic process (GO: 0044238) (Supplementary Figure S4). In the last layer, 60 genes involving drought (17), heat (11), combined drought and heat stress (17) and normal (11) conditions were enriched to regulation of transcription (GO: 0045449) (Supplementary Tables S1, S4 and Supplementary Figure S4). In addition, most of the 60 genes (41, 68.33%) were transcription factors, such as WRKY, bHLH, Zinc finger, bZip, *et al.*

The expression profiling analysis was used to assess the response of candidate genes to DS tolerance. Among the genes with expression profile successfully identified, 46 of them were determined as DEGs under DS *vs.* WW conditions (log_2_^foldchange^ > 1 and FDR < 0.01) in at least one line (Supplementary Table S4 and Figure 5B). Out of all the 46 DEGs, 16 (35%) were significantly associated with the traits under drought condition, and 12 (26%) were significantly associated with the traits under combined drought and heat stress condition (Supplementary Table S4). The expression profiles of the determined DEGs were highly consistent at different levels among the four maize lines with big difference on DS tolerance. In all the four maize lines, the expression levels of the candidate genes including GRMZM2G156861, GRMZM2G035153, and GRMZM2G094304 expressed under DS were much lower than those expressed under WW (fold change values < -1). The expression levels of some candidate genes including GRMZM2G103972, GRMZM2G016290, and GRMZM2G060544 expressed under DS were much higher than that expressed under the WW condition. In addition, two valuable DEGs (GRMZM2G429113 and GRMZM2G088613) were observed, the expression trends of these two DEGs was consistently expressed with the DS tolerance levels of the four tested maize lines. The above results indicated that the genes conserved in the four maize lines, plays an important role in the physical and biological process in response to drought tolerance.

### Genomic Prediction Under Different Conditions

GP for all the 12 trait-environment combinations were shown in Table 3, with different marker densities. The mean and range of *r_MG_* differed among the 12 trait-environment combinations under both marker densities. Under the same management condition, the *r_MG_* mean of the complex trait (GY and ASI) was consistently lower than that of the less complex trait (AD). For the same trait evaluated under the different management conditions, the *r_MG_* mean of the target trait evaluated under the WW condition was consistently higher than that evaluated under different stress conditions, i.e., DS, HS, and DHS.

**Table 3:** The genomic prediction accuracies (*r_MG_*) of the 12 trait-environment combinations using two kinds of marker density of marker-trait associated SNPs, and randomly selected SNPs filtered with minor allele frequency greater than 0.05 and 0% missing.

Trait-Environment^*^	Marker-trait associated SNPs				Randomly selected SNPs			
	Mean	Range	^¶^SD	^+^CV (%)	Mean	Range	^¶^SD	^+^CV (%)
GY-WW	0.75	0.53–0.87	0.06	7.39	0.59	0.35–0.77	0.08	13.85
GY-DS	0.43	0.11–0.71	0.13	29.02	0.50	0.23–0.68	0.09	18.58
GY-HS	0.69	0.49–0.86	0.06	9.36	0.38	0.04–0.70	0.12	30.31
GY-DHS	0.58	0.37–0.74	0.08	13.81	0.35	0.05–0.62	0.12	34.95
AD-WW	0.78	0.62–0.89	0.05	6.77	0.64	0.46–0.82	0.07	10.90
AD-DS	0.77	0.64–0.90	0.06	7.45	0.62	0.41–0.77	0.07	11.46
AD-HS	0.61	0.24–0.79	0.09	14.42	0.45	0.20–0.65	0.09	19.52
AD-DHS	0.72	0.54–0.84	0.07	9.12	0.51	0.22–0.72	0.10	19.78
ASI-WW	0.65	0.35-0.84	0.10	14.41	0.40	0.07–0.71	0.12	29.50
ASI-DS	0.62	0.39–0.81	0.08	12.83	0.55	0.21–0.77	0.10	18.48
ASI-HS	0.28	–0.21–0.62	0.15	50.96	0.13	–0.20–0.47	0.15	114.62
ASI-DHS	0.60	0.29–0.77	0.08	13.59	0.29	0.07–0.56	0.10	34.60

^*^The traits of GY, AD, and ASI are abbreviated from grain yield, anthesis date and anthesis–silking interval, respectively. The environments of WW, DS, HS and DHS are abbreviated from well-watered, drought stress, heat stress and combined drought and heat stress management conditions. ^¶^SD, standard deviation; ^+^CV, coefficient of variation.

The number of trait associated SNPs used for different trait-environment combination were ranged from 8 to 339, whereas for random genome-wide markers we used 10,108 high-quality SNPs. Interestingly, the mean accuracy was higher when the prediction was based on trait linked markers obtained through GWAS than the random whole genome wide high-quality SNPs for all 12 trait-environment combinations except for GY-DS. The average *r_MG_* value obtained from the trait associated SNPs was 0.75, 0.43, 0.69, and 0.58 for the GY evaluated under WW, DS, HS, and DHS condition, respectively. The corresponding *r_MG_* mean value obtained from the whole genome wide high-quality SNPs was 0.59, 0.50, 0.38, and 0.35 for the GY evaluated under WW, DS, HS, and DHS condition, respectively. Similar trend was also observed for AD and ASI evaluated under the different conditions. This result indicated that the trait associated SNPs corresponding to the target trait is more effective than the whole genome wide randomly selected SNPs for GP.

## Discussion

DS, HS, and DHS have been recognized as the major abiotic constraints to maize yields in the main production regions. Previous studies indicated that maize is highly susceptible to abiotic stresses during flowering time, secondary traits including AD and ASI, with strong genetic correlation with GY, are potential to be included in the breeding program to facilitate the effective selection on GY (Bennetzen and Hake, 2008; Lu et al., 2010). The information on phenotypic variation for GY and flowering time and revealing the correlations between the GY and the secondary traits, is helpful for understanding how to use secondary traits to facilitate the selection on GY. Results in the present study showed that the genetic diversity at phenotypic level for all the 12 trait-environment combinations is broad in the DTMA panel, and the heritability of all the target traits evaluated under the different conditions were moderate to high. These information is consistent with the results of the previous studies (Sivakumar et al., 2005; Cairns et al., 2013a), which indicated that the GY improvement through phenotypic selection is effective by reliable phenotypic evaluation. The phenotypic analysis also showed that GY was negatively and significantly correlated with ASI under each evaluation condition, this corroborates with the previous observation (Ribaut et al., 2009). It indicated that ASI is an appropriate secondary trait to facilitate the selection on GY.

The information of dissecting the genetic architecture of GY and secondary traits evaluated under different conditions and identifying their significantly associated important genomic regions is helpful in accelerating the efforts on rapid development of the stress-tolerant maize germplasm either through marker assisted selection and/or GS. The SNP-based association mapping results showed 1549 SNPs significantly associated with all 12 trait-environment combinations at the *p*-value threshold of 1 × 10^-4^. The average PVE of all the significant SNPs was 4.33%, and 541 of them had a PVE value greater than 5%. These observations indicate that the GY and secondary trait of flowering time under optimal and stress conditions are complex in nature, controlled by multiple minor QTL with small effects distributing across the maize genome. It also shown the difficulty of improving maize GY and the secondary trait of flowering time under the stress conditions by using marker assisted selection due to the genetic architecture complexity of the target traits. In addition, the previous phenotypic analysis also observed that the tolerance to DHS in maize was genetically distinct from tolerance to individual stresses, and tolerance to either stress alone did not confer tolerance to DHS (Cairns et al., 2013a). The SNP-based association mapping results observed in the current study are highly consistent with the observations of the phenotypic correlation analysis. Few SNPs and candidate genes were overlapped for the same trait under the different conditions. For the GY, no overlapping SNPs were observed among all the evaluation conditions, and one overlapping candidate gene each was observed between WW and DS conditions, and between WW and HS conditions. This information indicates the difficulty of improving maize GY simultaneously response to multiple abiotic stress tolerances.

Compared to single SNP-based association mapping, the haplotype-based association mapping detected fewer number of significant associations and candidate genes but with higher PVE for haplotypes, indicating the improved mapping power by employing the candidate-gene-haplotypes constructed with the GBS SNPs in the genic regions. The previous studies also showed that the GBS has emerged as a powerful tool for genetic diversity analysis, linkage mapping, GWAS and GS (Zhang et al., 2015; Wu et al., 2016; Cao et al., 2017), where the average linkage disequilibrium decay distance over all ten chromosomes in tropical association mapping panel was less than 5 kb at *r*^2^ = 0.1. The mapping power of the single SNP-based association mapping in the previous studies were improved due to the smaller linkage disequilibrium decay distance. The present study showed that both the single SNP-based and haplotype-based association mapping analyses are powerful for revealing the genetic architecture of the complex traits using high density GBS SNPs. In total, 673 and 19 candidate genes were identified from SNP-based and haplotype-based association mapping, respectively. Some of these candidate genes were validated by the expression profiling analysis. Four overlap candidate genes, i.e., GRMZM2G329229, GRMZM2G313009, GRMZM2G043764, and GRMZM2G109651, were observed in both the SNP-based association mapping and the haplotype-based association mapping analyses (Table 2 and Supplementary Table S3). These reliable candidate genes, with PVE of above 9.13%, will be further validated in the linkage mapping analysis and gene function and mechanism analysis.

Some candidate genes reported in the previous studies were also observed in the current study. In total, eight candidate genes related with GY (Salvi et al., 2007; Zhang et al., 2014; Liu et al., 2016), two candidate genes related with AD (Sawers et al., 2004; Hirsch et al., 2014), and one candidate genes related with ASI (van Nocker et al., 2000), were highlighted in Figure 3 and Supplementary Figures S1, S2, respectively. In the previous reported QTL interval of qKRN8-1 controlling kernel row number (Liu et al., 2016), several candidate genes significantly associated with GY were identified, i.e., candidate gene GRMZM2G331566 encoding glycosyl hydrolase 9B13 associated with GY-WW, serinc-domain containing serine and sphingolipid biosynthesis protein gene GRMZM2G088356 and ubiquitin-conjugating enzyme gene GRMZM2G007276 associated with GY-DH. Gene GRMZM2G700665 was associated with GY-WW, however, it was annotated as *ZmRap2.7*, a candidate gene involved in flowering time control (Salvi et al., 2007). In the previous reported QTL interval of qKRN1 at bin 1.10 controlling kernel row number, GRMZM5G881950, significantly associated with GY-WW, were identified (Liu et al., 2016). In the previous reported QTL interval on chromosome 3 effecting the maize kernel thickness, candidate gene GRMZM2G104081 encoding hexokinase 1, was identified and significantly associated with GY-WW (Zhang et al., 2014). For the target traits of AD and ASI, candidate gene GRMZM2G171650 and *ZmLD*, located on chromosome 3, was reported previously and significantly associated with AD-DH and ASI-WW, respectively. GRMZM2G171650, encoding MADS-box family protein with MIKC^*^ type-box and effecting flowering time, was previously identified in an association mapping analysis by Hirsch et al. (2014). *ZmLD*, encoding homeodomain-like superfamily protein, was identified by van Nocker et al. (2000), it expressed in the shoot apex and developing inflorescences in maize. *ZmHy2*, located on chromosome 8 and associated with AD-DH in the current study, was also reported in the previous studies (Sawers et al., 2002, 2004; Dong et al., 2012). This gene prevents the synthesis of the phytochrome chromophore 3*E*-phytochromobilin (PΦB), and effects the flowering time.

Besides the previous reported candidate genes, new candidate genes with different functions were also identified in the current study. In total, nine new candidate genes related with GY, 10 new candidate genes related with AD, and seven new candidate genes related with ASI, were highlighted in Figure 3 and Supplementary Figures S1, S2, respectively. GRMZM2G131611, encoding zinc finger protein, was significantly associated with GY-WW. GRMZM2G048733 (a regulatory component of ABA receptor) and GRMZM2G145458 encoding jasmonate-ZIM-domain protein, were significantly associated with GY-DS. GRMZM2G151863, encoding GDT1-like protein, was significantly associated with GY-DH with the most significant *p*-value. GRMZM2G068294, encoding phytochrome A-associated F-box protein, was associated with AD-WW, AD-DS and AD-HS simultaneously. *ZmGR2c*, associated with AD-DS, is a gibberellin responsive gene. Seven SNPs harbored by GRMZM5G866432 were associated with ASI-DS with average PVE value of 5.17%. WRKY DNA-binding protein gene GRMZM2G076657 was associated with ASI-DH.

Genomic selection incorporates all the available molecular marker information by capturing both the minor and major QTL of the target trait simultaneously. GS become an effective approach for complex trait improvement. In this study, the target traits of GY, AD and ASI are polygenic traits, and ASI and GY are more complex than AD, as demonstrated by the heritability (Cairns et al., 2013a; Zhang A. et al., 2017). In GS, the less complex trait AD had higher *r_GM_* compared to the other two traits, which is consistent with the nature of complexity (Zhang A. et al., 2017). Incorporating the trait associated markers into the prediction model has potential to improve the prediction accuracy of the complex traits as observed in this study. However, the prediction accuracy is possible to be overestimated, and further analysis have to be performed to validate this, when the training population and the breeding population are different.

## Data Archiving

The phenotypic data of the estimated best linear unbiased prediction values and the SNP data is available from the following repository: http://hdl.handle.net/11529/10548156. The raw RNA sequencing data is available from the following repository in the NCBI Sequence Read Archive under accession number PRJNA294848 (SRP063383): https://www.ncbi.nlm.nih.gov/bioproject/?term=PRJNA294848.

## Author Contributions

RB, MG, XZ, and MO coordinated the genotyping. JC, DM, CM, FSV, and BP coordinated the phenotyping. YY and YaL performed the RNA-seq. YY, AZ, YuL, NW, ZH, YaL, and XZ carried out the data analysis and wrote this manuscript.

## Conflict of Interest Statement

The authors declare that the research was conducted in the absence of any commercial or financial relationships that could be construed as a potential conflict of interest.

## Footnotes

1 https://www.r-project.org/

2 www.panzea.org

3 http://www.maizegdb.org/

4 https://sourceforge.net/projects/tassel/files/Tassel%203.0/2013-04-25/

5 https://blast.ncbi.nlm.nih.gov/Blast.cgi

6 https://phytozome.jgi.doe.gov/pz/portal.html#!info?alias=Org_Zmays

7 http://bioinfo.cau.edu.cn/agriGO/index.php

## Supplementary Material

FIGURE S1 The Manhattan plots and Q-Q plots of the SNP-based (gray dots) and the haplotype-based (green and yellow dots) association mapping for AD under different conditions. WW, DS, HS, and DHS are abbreviated from well-watered, drought stress, heat stress and combined drought and heat stress management conditions. The red colored candidate genes are the previously reported, and the blank colored are novel candidate genes.

FIGURE S2 The Manhattan plots and Q-Q plots of the SNP-based (gray dots) and the haplotype-based (green and yellow dots) association mapping for ASI evaluated under different conditions. WW, DS, HS, and DHS are abbreviated from well-watered, drought stress, heat stress and combined drought and heat stress management conditions. The red colored candidate genes are the previously reported, and the blank colored are novel candidate genes.

FIGURE S3 The genome-wide landscape of significant SNPs (blue and red vertical lines) for the 12 trait-environment combinations. The red lines indicate the SNPs are in hotspot regions, where the thresholds are shown in Supplementary Table S2. The light and dark gray colored rectangles are chromosomes and the corresponding centromeres, respectively. GY, AD, and ASI indicate grain yield, anthesis date and anthesis-silking interval, respectively. WW, DS, HS, and DHS indicate well-watered, drought stress, heat stress and combined drought and heat stress management conditions.

FIGURE S4 Hierarchical tree graph of overrepresented gene ontology (GO) terms in biological process category generated by singular enrichment analysis. Boxes in the graph represent GO terms labeled by their GO ID, term definition and statistical information. The significant (adjusted *p* ≤ 0.05) and non-significant terms are marked with color and white boxes, respectively. The diagram, the degree of color saturation of a box is positively correlated to the enrichment level of the term. Solid, dashed, and dotted lines represent two, one, and zero enriched terms at both ends connected by the line, respectively. The rank direction of the graph is set to from left to right.

TABLE S1 The SNP-based genome-wide association mapping results of the 12 trait-environment combinations in the DTMA panel.

TABLE S2 The hotspot information of the significantly associated SNPs of all the 12 target-environment combinations in the DTMA panel.

TABLE S3 The haplotype-based genome-wide association mapping results of the 12 target trait-environment combinations in the DTMA panel.

TABLE S4 The log2 fold-change (WS/WW) of the differentially expressed candidate genes identified in association mapping analysis in four maize lines with different drought tolerance.
