# Supplementary figures and images for: Genome-Wide Association Mapping and Genomic Prediction Analyses Reveal the Genetic Architecture of Grain Yield and Flowering Time Under Drought and Heat Stress Conditions in Maize
Source: Front Plant Sci. 2019 Jan 30;9:1919. doi: 10.3389/fpls.2018.01919 (PMC6363715; doi:10.3389/fpls.2018.01919)

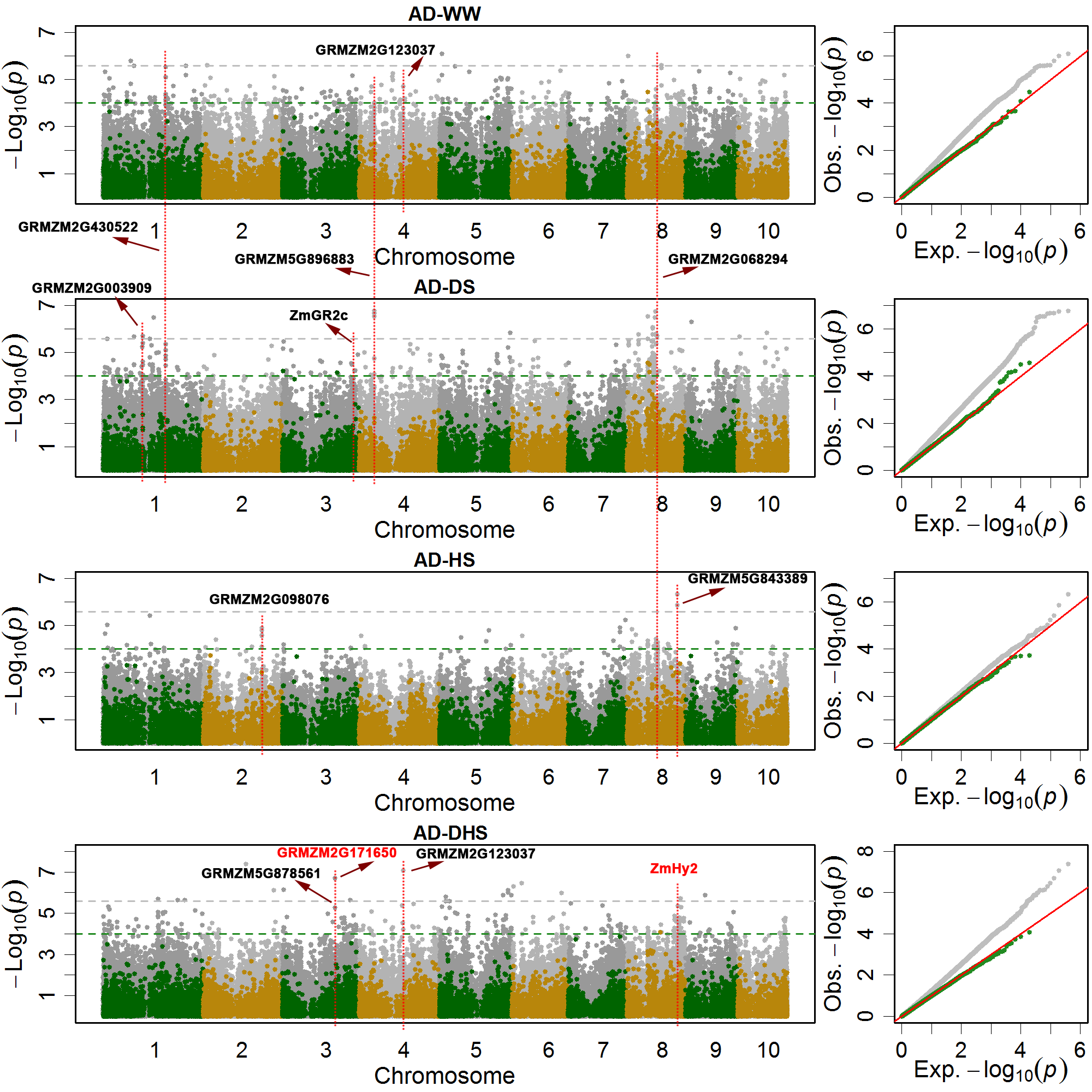

Supplement: FIGURE S1 — The Manhattan plots and Q-Q plots of the SNP-based (gray dots) and the haplotype-based (green and yellow dots) association mapping for AD under different conditions. WW, DS, HS, and DHS are abbreviated from well-watered, drought stress, heat stress and combined drought and heat stress management conditions. The red colored candidate genes are the previously reported, and the blank colored are novel candidate genes. [file Image_1.TIF]

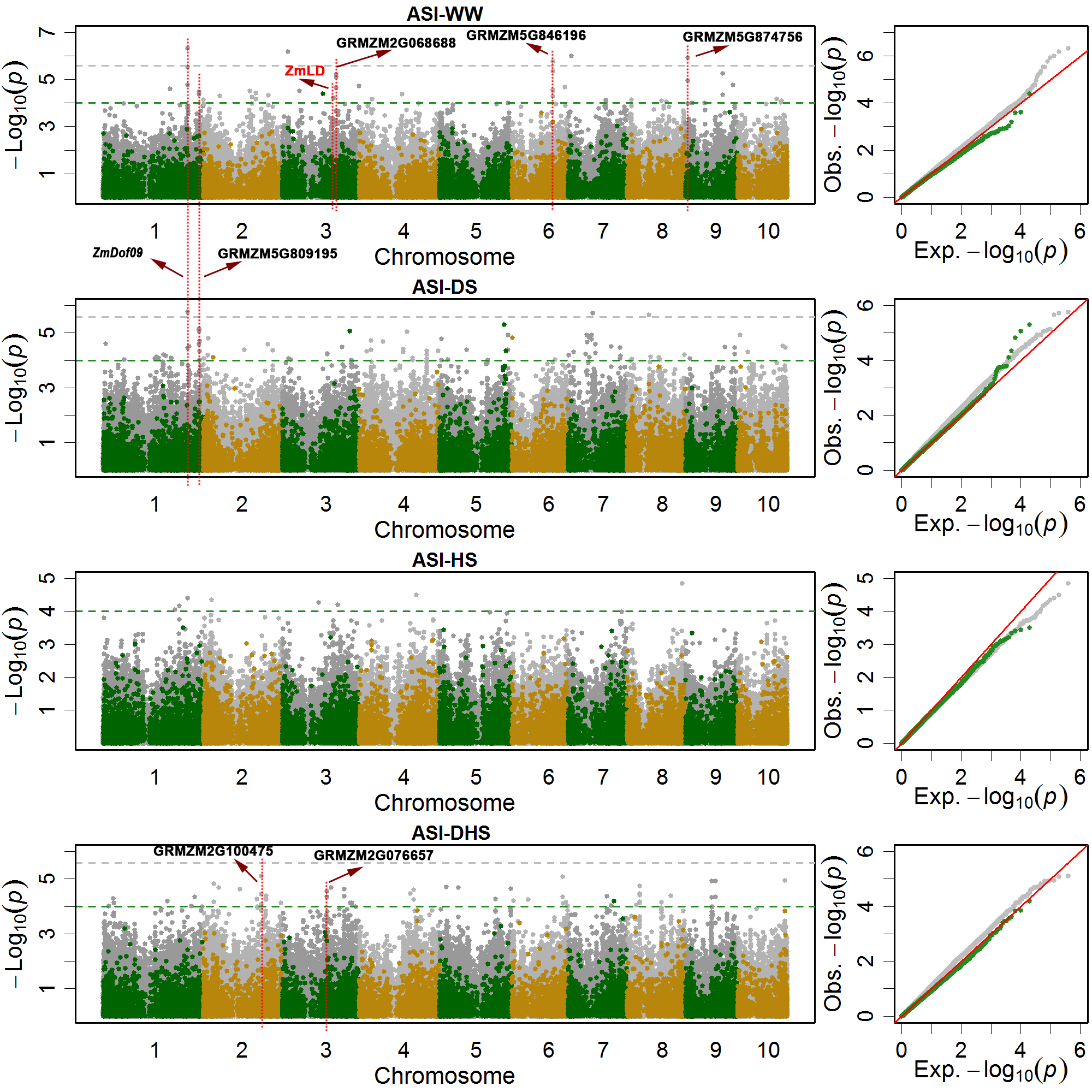

Supplement: FIGURE S2 — The Manhattan plots and Q-Q plots of the SNP-based (gray dots) and the haplotype-based (green and yellow dots) association mapping for ASI evaluated under different conditions. WW, DS, HS, and DHS are abbreviated from well-watered, drought stress, heat stress and combined drought and heat stress management conditions. The red colored candidate genes are the previously reported, and the blank colored are novel candidate genes. [file Image_2.TIFF]
